# The role of juvenile hormone and insulin/TOR signaling in the growth of *Manduca sexta*

**DOI:** 10.1186/s12915-015-0155-z

**Published:** 2015-06-25

**Authors:** Nicole E. Hatem, Zhou Wang, Keelin B. Nave, Takashi Koyama, Yuichiro Suzuki

**Affiliations:** Department of Biological Sciences, Wellesley College, 106 Central St., Wellesley, MA 02481 USA; Development, Evolution and the Environment Lab, Instituto Gulbenkian de Ciência, 2780-156 Oeiras, Portugal

**Keywords:** Developmental timing, Critical weight, Life history trade-offs, *Manduca sexta*, *Drosophila melanogaster*, Metamorphosis, Insulin/TOR signaling, Juvenile hormone, Prothoracic gland, Molt timer

## Abstract

**Background:**

In many insect species, fitness trade-offs exist between maximizing body size and developmental speed. Understanding how various species evolve different life history strategies requires knowledge of the physiological mechanisms underlying the regulation of body size and developmental timing. Here the roles of juvenile hormone (JH) and insulin/target of rapamycin (TOR) signaling in the regulation of the final body size were examined in the tobacco hornworm, *Manduca sexta*.

**Results:**

Feeding rapamycin to wild-type larvae decreased the growth rate but did not alter the peak size of the larvae. In contrast, feeding rapamycin to the JH-deficient *black* mutant larvae caused the larvae to significantly increase the peak size relative to the DMSO-fed control animals by lengthening the terminal growth period. Furthermore, the critical weight was unaltered by feeding rapamycin, indicating that in *Manduca*, the critical weight is not influenced by insulin/TOR signaling. In addition, post-critical weight starved *black* mutant *Manduca* given rapamycin underwent metamorphosis sooner than those that were fed, mimicking the “bail-out mechanism”.

**Conclusions:**

Our study demonstrates that JH masks the effects of insulin/TOR signaling in the determination of the final body size and that the critical weights in *Drosophila* and *Manduca* rely on distinct mechanisms that reflect different life history strategies. Our study also suggests that TOR signaling lengthens the terminal growth period in *Manduca* as it does in *Drosophila*, and that JH levels determine the relative contributions of nutrient- and body size-sensing pathways to metamorphic timing.

## Background

Growth control has important consequences for an organism’s fitness [1]. Body size can impact the organism’s reproductive output as well as performance. Developmental time also has important consequences for an organism’s fitness, especially in geographic locations with seasonality. These two aspects of growth, however, exhibit fitness trade-offs: maximizing body size tends to lengthen the developmental time, whereas accelerating developmental time necessitates the truncation of the growth period, resulting in smaller adult forms. Selection therefore molds body size control and developmental time regulation in a taxon-specific manner [2]. However, the degree of conservation in the underlying mechanisms remains unclear.

The physiological control of body size has been well studied in the tobacco hornworm, *Manduca sexta*. In *Manduca*, metamorphosis is triggered when ecdysteroid titers surge in the final instar, and the final body size is determined by a combination of factors that regulate the timing of ecdysteroid secretion. A key factor is the attainment of a size assessment point known as the critical weight. The final body size is also influenced by the time between the critical weight attainment and ecdysteroid surge, which is known as the terminal growth period [3]. In addition, growth rates influence the final body size. Using these parameters, the final body size of the larvae can be predicted accurately [4].

The critical weight is the size during the final instar when starvation no longer delays metamorphosis. Until the larva reaches the critical weight, the brain of the final instar larva is unable to secrete prothoracicotropic hormone (PTTH), which is necessary for the production and release of ecdysteroids from the prothoracic glands [5]. The key hormone that inhibits PTTH release from the brain is juvenile hormone (JH), a sesquiterpenoid hormone. Once the critical weight is reached, JH is actively cleared from the hemolymph, allowing the brain to release PTTH and stimulate ecdysteroid release [6]. The surge in the hemolymph ecdysteroids then triggers a series of morphological and behavioral sequences that mark the onset of metamorphosis. In *Manduca*, the critical weight is a time point when an unknown mechanism initiates the clearing of JH from the hemolymph. Oxygen level has been proposed to impact the timing of critical weight. When larvae are reared in hypoxic conditions, they attain critical weight at a smaller size than those reared at normoxic conditions, suggesting that the body senses oxygen levels to assess its body size [7]. Note that the critical weight itself is not influenced by JH but appears to be determined by the size at the onset of the final instar [4, 8]. Thus, the size at the onset of the final instar predicts the critical weight.

In *Drosophila,* starvation early in the final instar also leads to a delay in metamorphosis [9, 10]*.* When the mass at starvation is graphed against the time to metamorphosis, the slope of the regression line changes. This inflection point has been identified as the critical weight in *Drosophila* [10, 11]. The extent to which this critical weight is analogous to that seen in *Manduca* is not well understood, but there are a few key differences. Firstly, the critical weight in *Drosophila* occurs within the first 12 h after ecdysis to the final instar larva (about 25 % into the third instar), whereas in *Manduca*, a size assessment point is reached much later (about 50 % into the final instar) [11]. Secondly, starvation post critical weight in *Drosophila* causes the larva to accelerate the time to metamorphosis [10, 12], whereas in *Manduca*, post critical weight starved larvae initiate metamorphosis at the same time as normally fed larvae [5]. However, similar to *Manduca*, the clearing of JH appears to be an event associated with the attainment of critical weight: allatectomized larvae, which have the source of JH surgically removed, pupariate sooner than wild-type larvae despite the fact that the critical weight is not altered [13].

Recent studies in *Drosophila* have provided a genetic perspective on the control of metamorphic timing. In particular, the role of insulin/target of rapamycin (TOR) signaling has been demonstrated to play a major role in the timing of metamorphosis [12, 14, 15]: enlarging the prothoracic gland by increasing insulin signaling leads to precocious release of ecdysteroids and an accompanying reduction in the adult body size, whereas decreasing insulin signaling in the prothoracic glands leads to larger body size by delaying the release of ecdysteroids [12]. This effect has been interpreted to reflect the regulation of critical weight [12]. More recently, Foxo, a downstream target of insulin/TOR signaling, has been shown to regulate the critical weight by influencing the timing of the shift in the response to starvation [11]. In addition, TOR signaling has been shown to regulate the terminal growth period [14]. Inhibition of TOR signaling in the prothoracic glands by activating the TOR inhibitor tuberous sclerosis complex (TSC1/2) [16] leads to a reduction in ecdysteroid biosynthesis and, consequently, the lengthening of the terminal growth period [14]. The role of insulin/TOR signaling in the growth and size determination of *Manduca* larvae, however, remains unclear. The role of insulin signaling on ecdysteroid biosynthesis in cultured *Manduca* prothoracic glands appears to be minimal, although the role of TOR signaling has not been examined [17].

In penultimate instar *Manduca* larvae, TOR signaling has been shown to play an important role in ecdysteroid biosynthesis/release [18], and in the final instar *Manduca*, allatectomized larvae have been shown to have an amino acid-dependent timer that dictates the timing of the metamorphosis [19]. Thus, insulin/TOR signaling may also play a key role in the final instar *Manduca* larvae. To determine the relative contribution of JH and insulin/TOR signaling in the determination of the timing of the metamorphic onset, and to delineate the similarities and differences in the regulation of growth between *Drosophila* and *Manduca*, we compared the responses of wild-type and JH-deficient *black* mutant *Manduca* larvae to rapamycin. Rapamycin is an inhibitor of TOR signaling that also causes a reduction of insulin signaling because of their considerable crosstalk [20]. The *black* mutant has a mutation in the JH biosynthetic pathway that causes the adult size to be halved [4, 21]. Our findings suggest that both a body size-sensing pathway mediated by JH and a nutrition-sensitive pathway mediated by insulin/TOR signaling exist in *Manduca* and that JH normally masks the nutrition-sensitive pathway to determine the timing of metamorphosis. We further show and discuss that the critical weight in *Manduca* and the critical weight in *Drosophila* are regulated by distinct mechanisms even though they are operationally defined in the same manner.

## Results

### Rapamycin increases the peak body size of *black* mutant larvae but not wild-type larvae

**Fig. 1:**
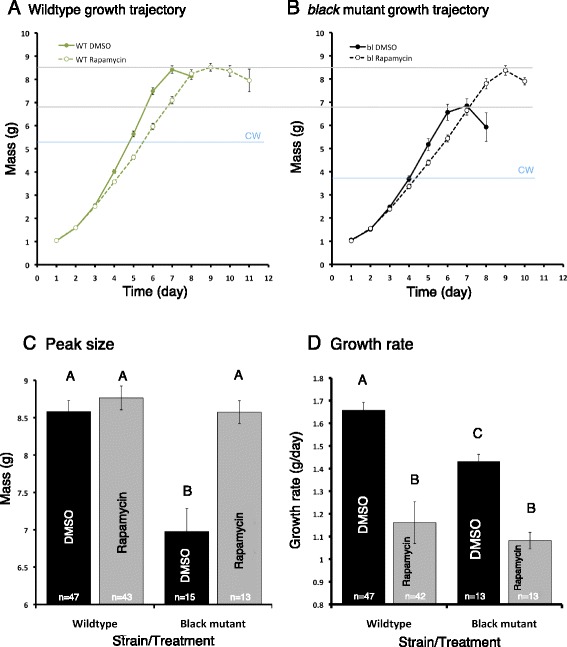
Growth trajectories of wild-type and *black* mutant fifth instar *Manduca* larvae fed rapamycin or DMSO. **a**, **b** The growth trajectories of the wild-type (**a**) and the *black* mutant (**b**) larvae. The solid line with closed circles is the control animals and the dashed line with open circles is the rapamycin-treated animals. Blue lines represent critical weights as determined in Fig. 3. **c**, **d** The average peak masses (**c**) and growth rates (**d**) of the two strains raised on different diet treatments. Gray bars represent animals reared on diets with rapamycin, and black bars represent those reared on diets with DMSO. Bars with different letters represent statistically significant differences (one-way ANOVA, *P* < 0.001 for the peak masses and *P* < 0.0001 for growth rates). Error bars represent standard errors

In order to determine the effect of inhibiting insulin/TOR signaling in the wild-type and the JH-deficient *black* mutant larvae, fifth instar larvae were fed either a diet treated with rapamycin or dimethyl sulfoxide (DMSO). The average time to gut purge from the onset of feeding was significantly longer for the wild-type larvae fed rapamycin than those fed DMSO (mean of 7.8 days as opposed to 5.7 days; one-way ANOVA, post hoc Tukey test, *P* = <0.0001), and the growth rate was slower in the rapamycin group (mean of 1.65 g/day as opposed to 1.16 g/day) (one-way ANOVA, post hoc Tukey test, *P* = <0.0001) (Fig. 1a, d). The rapamycin-fed *black* mutant larvae also took significantly longer to gut purge than those fed the control diet (mean of 8.1 days as opposed to 5.5 days; one-way ANOVA, post hoc Tukey test, *P* = <0.0001) and had a slower growth rate (mean of 1.08 g/day as opposed to 1.43 g/day) (one-way ANOVA, post hoc Tukey test, *P* = <0.001). Although the growth rate and duration were altered in response to rapamycin treatment, there was no significant difference between the peak weights of the wild-type larvae regardless of diet condition (Fig. 1a, c) (one-way ANOVA, post hoc Tukey test, *P* = 0.825). In contrast, the rapamycin-fed *black* mutant larvae attained a significantly larger peak size (one-way ANOVA, post hoc Tukey test, *P* < 0.05), growing more slowly but for a longer time period (Fig. 1).

**Fig. 2:**
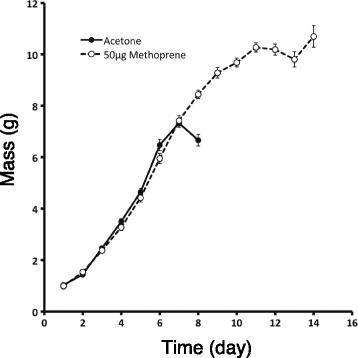
Effect of methoprene application on the growth of *black* mutant larvae. Larvae were treated with either 50 μg methoprene (dashed line with open circles) or acetone (solid line with closed circles) and weighed daily

Interestingly, the peak sizes of the rapamycin-treated wild-type and *black* mutant larvae were similar (approximately 8.6 g; one-way ANOVA, post hoc Tukey test, *P* = 0.932) (Fig. 1a, b). We therefore wondered if this was due to the *black* mutant larvae reaching a maximum size caused by a growth constraint imposed by the exoskeleton. Thus, we treated *black* mutant larvae with the JH mimic methoprene, which delays gut purge in wild-type larvae and causes them to grow until a limit is reached [4]. Methoprene-treated *black* mutant larvae exhibited a growth trajectory similar to that of acetone-treated control larvae but continued to grow to approximately 10.6 g (Fig. 2). Thus, the peak weight attained by rapamycin-treated animals does not represent a physical maximum size.

### Disrupting insulin/TOR signaling does not affect the critical weights in *Manduca* larvae but accelerates time to metamorphosis in starved *black* mutant larvae

Nijhout and Williams [5] first identified the critical weight using a graphical approach by drawing regression lines to describe the relationship between mass (on the x-axis) and time to the onset of metamorphosis (on the y-axis) in animals continuously fed or starved at particular masses. A single regression line can be fitted to describe the relationship between mass and time to metamorphosis in the continuously fed animals. The relationship between mass and time to metamorphosis for the starved animals is better described as two different regression lines. In wild-type *Manduca,* once the critical weight is attained, the lines for the starved animals and normal animals overlap and no difference is observed between starved and fed animals.

**Fig. 3:**
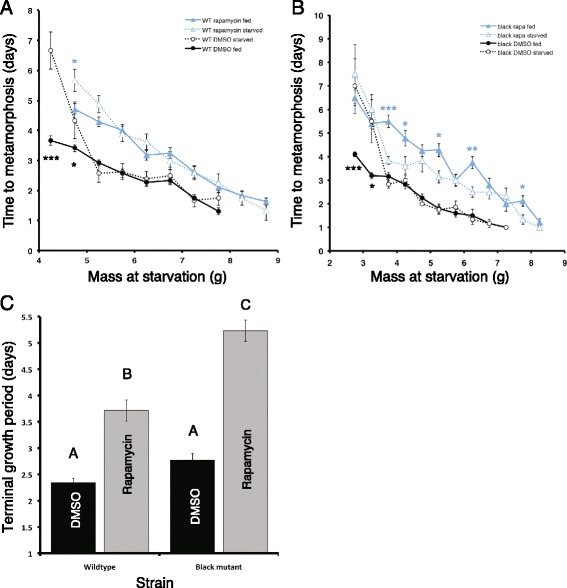
Critical weight determination of wild-type and *black* mutant larvae on rapamycin-treated diets. **a**, **b** Critical weight determination of wild-type (**a**) and *black* mutant larvae (**b**) on DMSO- or rapamycin-treated diets. The x-axis indicates the weight at which the animals were switched from the nutritive diet to the non-nutritive diet, and the y-axis represents the time from starvation to gut purge (measured in days). Black and blue lines represent animals fed a DMSO- or a rapamycin-treated diet, respectively. The solid lines and closed symbols represent larvae that were not starved, and the dotted lines and open symbols represent starved animals. Student’s *t*-test was used to compare the means for each weight category. *denotes *P* < 0.05; **denotes *P* < 0.001; ***denotes *P* < 0.0001. **c** The terminal growth period of wild-type and *black* mutant larvae on DMSO- or rapamycin-treated diets. Error bars represent standard errors

To determine whether the critical weight of *Manduca* is affected by feeding rapamycin, wild-type and *black* mutant larvae were either continuously fed or starved once they reached a particular mass. We found that the critical weight of wild-type larvae is larger than that of the *black* mutant larvae, as previously determined [4]. The critical weight was similar in both the rapamycin and DMSO treatment groups for the wild type (5–5.5 g, represented as 5.25 g on Fig. 3a). In the *black* mutants, DMSO-fed larvae had a critical weight of 3.5–4 g (represented as 3.75 g on Fig. 3b), similar to that reported for the untreated *black* mutant larvae [4]. Interestingly, in rapamycin-treated larvae, the starved larvae gut purged sooner relative to the fed animals. When starved at weight ranges smaller than 3.5 g, there was no significant difference in the time to gut purge between the starved and continuously fed larvae. In contrast, when larvae were starved above 3.5 g, the starved larvae tended to accelerate the time to gut purge (Fig. 3b). This shift coincides with the critical weight observed in the DMSO-fed larvae. We therefore infer that the critical weight is also 3.5–4 g for the rapamycin-treated larvae (Fig. 3b). Thus, feeding rapamycin did not alter the critical weight for both the wild-type and the *black* mutant larvae. Furthermore, diminished JH and insulin/TOR signaling caused starved animals to initiate metamorphosis sooner.

We next determined the terminal growth period by calculating the time from the attainment of the critical weight to gut purge. In both the wild-type and the *black* mutant larvae, the terminal growth period was significantly lengthened when rapamycin was added to their diet (Fig. 3c) (one-way ANOVA, post hoc Tukey test, *P* = <0.0001 for both strains). However, the terminal growth period was significantly longer in the *black* mutants fed a rapamycin-treated diet than the wild-type larvae fed the same diet (one-way ANOVA, post hoc Tukey test, *P* = <0.0001), even though the terminal growth periods for the two strains were similar for DMSO treatments (one-way ANOVA, post hoc Tukey test, *P* = 0.235). As shown above, the peak size of the rapamycin-treated *black* mutant larvae was significantly larger than that of the DMSO-treated *black* mutant larvae. Since the critical weight is similar for both the rapamycin and DMSO-treated animals, the increase in the peak size must result from the increase in the terminal growth period.

### Feeding rapamycin reduces phospho-4E-BP and phospho-Akt levels

**Fig. 4:**
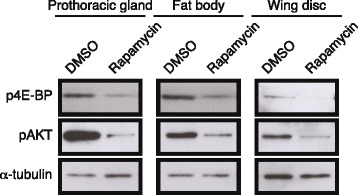
Effect of rapamycin on phospho-4E-BP and phospho-Akt expression in the fat body, wing discs, and prothoracic glands of wild-type and *black* mutant larvae. Tissues were pooled from 10 fifth instar larvae fed DMSO or rapamycin for one day. α-Tubulin was used as a loading control

To determine how rapamycin affects TOR and insulin signaling in different tissues, we isolated wing discs, fat body, and prothoracic glands from fifth instar larvae fed either a rapamycin-treated or DMSO-treated diet and examined phospho-4E-BP, a downstream component of TOR signaling, and phospho-Akt, a downstream component of insulin signaling. In all tissues, we found that the level of phospho-4E-BP was decreased in the rapamycin-treated larvae relative to those fed a DMSO-treated diet (Fig. 4), indicating that feeding rapamycin inhibits TOR signaling in all of these tissues. Similarly, the expression of phospho-Akt was also inhibited in rapamycin-treated larvae (Fig. 4), indicating that rapamycin also results in inhibiting insulin signaling in all of the tissues.

### The presence of wing discs or feeding a sugar-only diet does not delay the timing of metamorphosis

**Fig. 5:**
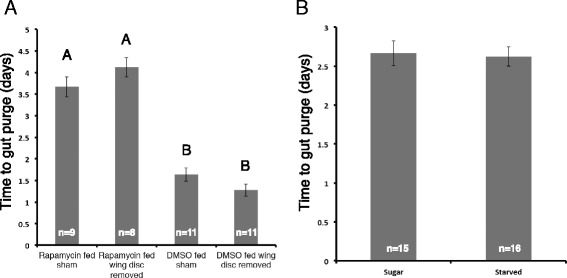
The effect of imaginal disc removal and sugar diet on metamorphic timing in *black* mutants. **a** The effect of wing imaginal disc removal on the time to gut purge in *black* mutant larvae fed diets with DMSO or rapamycin. Sham represents control for physical removal of imaginal discs. Different letters indicate statistically significant difference (one-way ANOVA, post hoc Tukey test, *P* < 0.0001). **b** Effect of sugar or starved diets on time to gut purge in *black* mutant larvae fed rapamycin. Error bars represent standard errors

We next explored potential mechanisms underlying the delayed onset of metamorphosis in feeding *black* mutant larvae given a rapamycin diet relative to the starved larvae. Colombani et al. and Garelli et al. found that, in *Drosophila*, the secreted factor dILP8 from growing or regenerating imaginal discs can delay release of ecdysteroids from the prothoracic glands [22, 23]. In order to determine whether there was a similar mechanism behind the delay in metamorphosis in fed *black* mutant *Manduca*, wing imaginal discs were removed from *black* mutant larvae, which were subsequently given rapamycin- or DMSO-treated diets. Larvae fed a rapamycin-treated diet took significantly longer to gut purge than animals fed DMSO (Fig. 5). However, there was no significant difference in the time to gut purge between larvae without wing imaginal discs and sham control larvae fed the same diet. Thus, growing imaginal discs do not appear to cause the delay observed in fed *black* mutant larvae.

Since TOR is an amino acid-sensitive pathway, we next explored whether sugar alone might impact the time to metamorphosis. However, no difference was observed between the larvae fed a sugar diet and those given a non-nutritive diet. Thus, sugar alone does not account for the delayed onset of metamorphosis seen in the feeding *black* mutant given a rapamycin diet.

### TOR signaling inhibition affects the prothoracic gland size in wild-type *Manduca* larvae

**Fig. 6:**
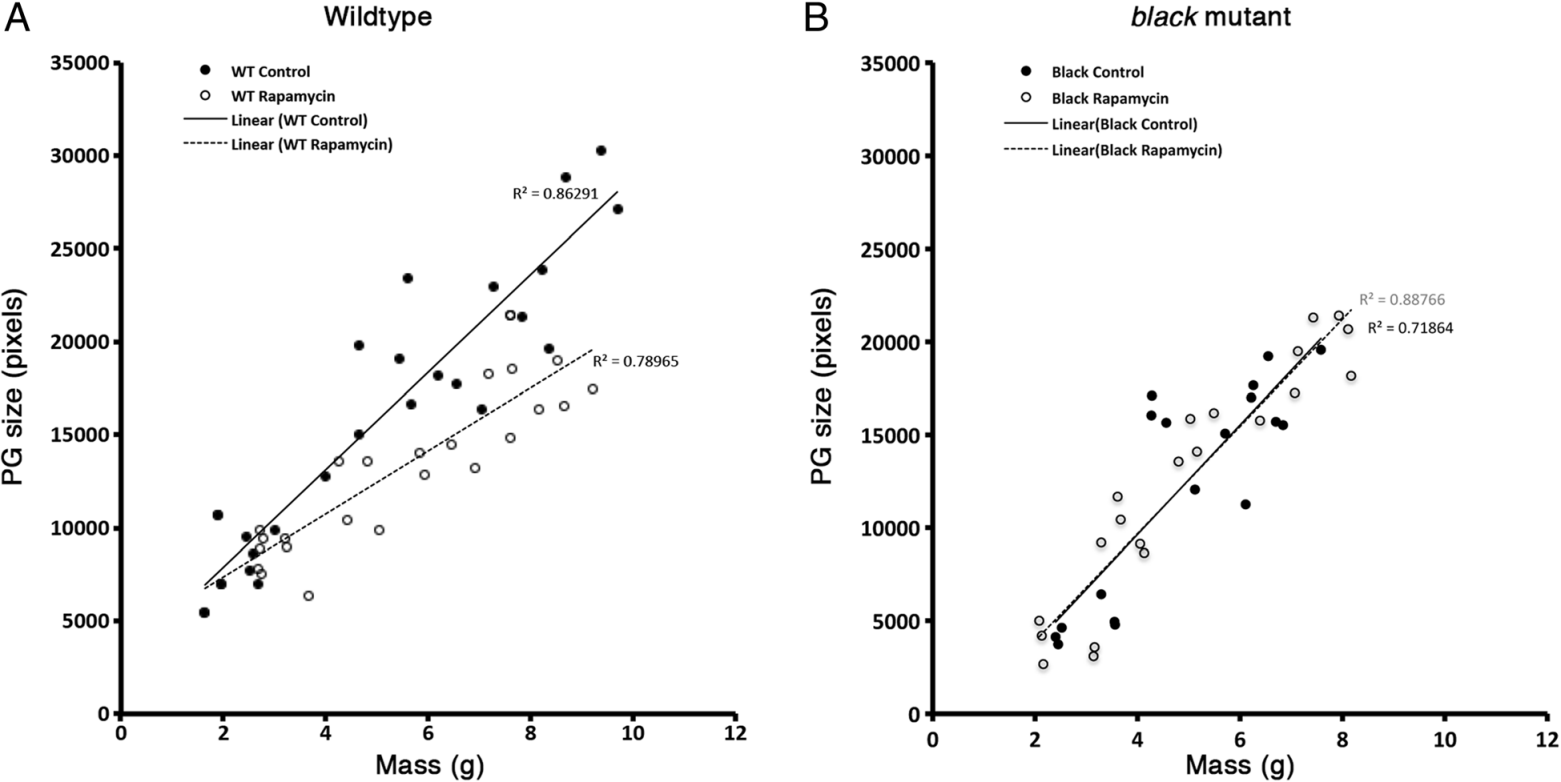
The relative growth of the prothoracic gland. **a**, **b** The growth of the prothoracic gland relative to body size in wild-type (**a**) and *black* mutant (**b**) *Manduca* fed diet with DMSO (solid line) or rapamycin (dotted line)

To determine whether the prothoracic gland size was affected by feeding rapamycin, prothoracic glands from larvae fed on rapamycin or DMSO were dissected at various body sizes. The prothoracic gland sizes of the rapamycin-fed wild-type animals relative to the body size were smaller than those of their DMSO-fed counterparts (Fig. 6a). At the critical weight of 5.25 g, a clear difference in the prothoracic gland sizes was observed. Thus, there was no correlation between the critical weight and prothoracic gland size in the wild-type larvae. In contrast, in the *black* mutant larvae, the prothoracic gland size varied with body size similarly between larvae fed a rapamycin diet and those fed a DMSO diet (Fig. 6b). Thus, at the critical weight, the prothoracic gland sizes of the *black* mutant larvae were identical regardless of whether they had been fed rapamycin.

## Discussion

### The effects of insulin/TOR signaling on the final body size are masked by JH in the final instar

In this study, we have found that feeding rapamycin leads to a delay in the timing of metamorphosis in both wild-type and *black* mutant *Manduca*. However, the peak body size of wild-type larvae is unaltered by rapamycin, whereas *black* mutant larvae fed rapamycin grow to a significantly larger body size. Ecdysteroid biosynthesis has been shown to be impacted by TOR signaling in *Manduca* [18]. Furthermore, our findings show that by feeding rapamycin, the timing of initiation of metamorphosis is delayed, indicating that the timing of ecdysteroid release is impacted by TOR signaling. However, the presence or absence of JH determines whether the final size is impacted by the nutrient-sensitive pathways: in the wild-type *Manduca*, the JH-mediated delay masks the effects of the nutrition-sensitive insulin/TOR signaling pathway, and the final size of the animal is not influenced by nutrient-sensitive pathways. In contrast, when JH titers are low, inhibition of the insulin/TOR signaling pathway can significantly delay the timing of metamorphosis and impact the final body size. Our findings thus suggest that JH acts to protect larvae from nutritional disturbances. In animals with higher JH titers, fluctuations in nutritional-sensing pathways have minimal effects on the final body size, whereas in low JH animals, fluctuations in nutrient-sensing pathways can impact the final body size significantly.

In contrast, in *Drosophila*, the insulin signaling pathway directly impacts the timing of ecdysteroid biosynthesis at critical weight [11]. Furthermore, the terminal growth period has been shown to be lengthened by inhibition of TOR signaling in the prothoracic glands [14]. Thus, in *Drosophila*, the timing of metamorphosis relies primarily on nutritional inputs that directly modulate ecdysteroid biosynthesis.

**Fig. 7:**
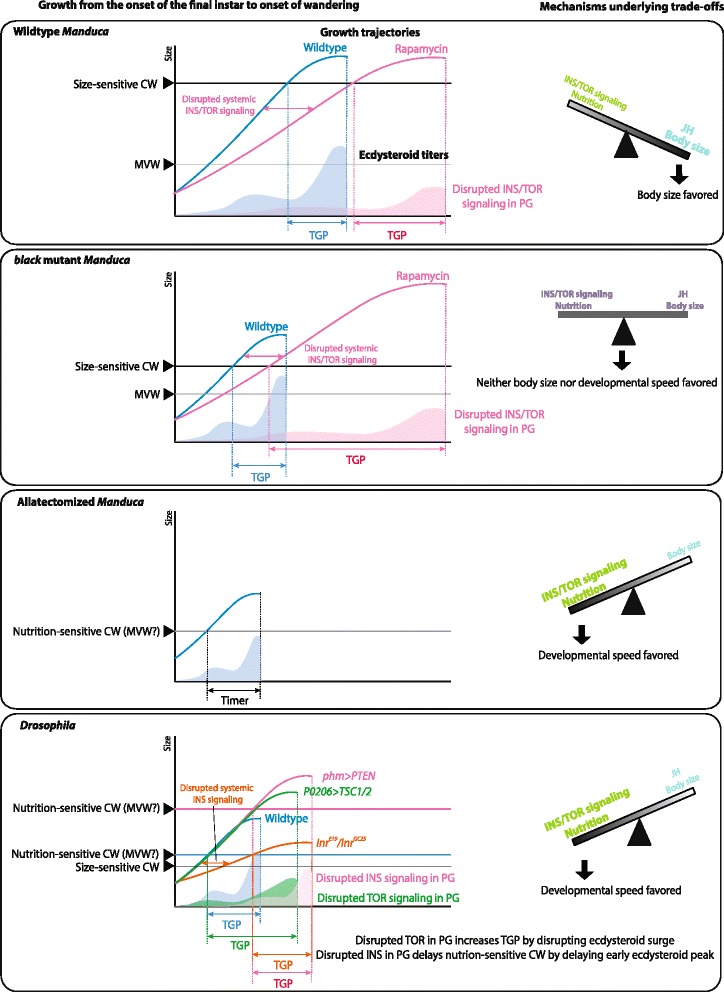
A model showing the effects of JH and insulin/TOR signaling on the timing of metamorphosis. (Left) The attainment of nutrition-sensitive critical weight is influenced by insulin signaling in the prothoracic glands [12], whereas the attainment of the size-sensitive critical weight determines the time when JH is cleared from the hemolymph [5]. Depending on the time when the size-sensitive critical weight is attained, the relative contribution of insulin signaling on the final body size changes. The model shows how JH and insulin/TOR signaling influence the growth of wild-type, *black* mutant, and allatectomized *Manduca*, and *Drosophila* [12, 14, 19]*.* (Right) The seesaw diagrams represent the mechanisms underlying trade-offs between maximizing body size and faster development. The relative contributions of JH signaling and insulin/TOR signaling pathways differ between *Manduca* and *Drosophila*: maximal body size is favored in *Manduca,* whereas developmental speed is favored in *Drosophila*

Since rapamycin significantly delays the timing of ecdysteroid secretion in the *black* mutant *Manduca* and impacts the final body size, we think that insulin/TOR signaling also regulates the terminal growth period in the *black* mutants. In this case, the JH-mediated delay period is removed, and the final body size of the larva reflects a nutrition-sensitive pathway. Our study therefore shows that insulin/TOR signaling and JH signaling provide two distinct mechanisms for determining the final size of the larva and that, depending on the species, one or the other mechanism dominates. We can therefore envision a seesaw with either JH or insulin/TOR signaling dominating to regulate the timing of metamorphic onset (Fig. 7, right). In wild-type *Manduca*, JH clearly dominates and determines the timing of metamorphosis based on body size. In contrast, in *Drosophila*, insulin/TOR signaling dominates to set the timing of metamorphosis based on nutritional intake. In *black* mutant larvae, we presume that the roles of JH and insulin/TOR signaling are more or less equal, because the larvae still have some JH but inhibiting insulin/TOR signaling delays the timing of metamorphosis. Thus, neither body size nor nutritional intake dominates.

In penultimate instar *Manduca* larvae, insulin/TOR signaling plays a critical role in the timing of molting [18]. When larvae are fed rapamycin, ecdysteroid titers are dramatically suppressed and the initiation of a molt is delayed, so that the larva grows to a larger size before molting. Since the molt of a penultimate instar larva is relatively unaffected by JH [24], ecdysteroid production is purely dependent on the rate of ecdysteroid biosynthesis. This situation would be similar to what is observed in the final instar *Drosophila* and JH-deficient *Manduca.* In contrast, in the wild-type *Manduca,* ecdysteroid release is dependent on JH because the PTTH secretion from final instar brains becomes sensitive to JH [5, 25]. Thus, JH in the final instar wild-type *Manduca* plays a much more predominant role in regulating developmental transitions than in the earlier instars or in *Drosophila*.

### The critical weight is not influenced by insulin/TOR signaling

We found that feeding rapamycin does not influence the critical weight in both wild-type and *black* mutant larvae. Our observations corroborate a previous finding that in wild-type *Manduca* the critical weight is regulated by a size-sensing pathway instead of a nutrient-sensing pathway. This size-sensing mechanism has been linked to oxygen levels [7]. In response to this size assessment cue, JH is cleared from the hemolymph, triggering the release of PTTH from the brain.

In contrast, in *Drosophila*, the critical weight is not linked to a body size-sensing mechanism. Instead, the critical weight is marked by a small increase in the ecdysteroid titer, which in turn is controlled by a nutrient-sensitive pathway [11, 26, 27]. Clearly, the underlying primary mechanism of critical weights differs between the two species. A previous study has shown that allatectomized *Manduca* larvae behave similarly to *Drosophila* in that if starved within the first 12 h of molting, the time of gut purge is delayed, whereas starvation after 24 h feeding no longer delays metamorphosis [28]. This switch in the response to starvation in the JH-less state is sensitive to a nutrition-dependent pathway and is similar to the situation in *Drosophila*. In contrast, in both the wild-type and *black* mutant *Manduca*, the critical weight observed is a distinct phenomenon and is clearly independent of the insulin/TOR signaling pathway.

The prothoracic gland size, however, was influenced by the depression of insulin/TOR signaling. Interestingly, in the wild-type *Manduca*, the prothoracic gland size at critical weight was drastically different between the rapamycin-fed and DMSO-fed animals (Fig. 6). It has previously been shown that the prothoracic glands could be an indicator for size in *Drosophila*. The prothoracic gland reaching a certain size is thought to act as a proxy for the overall body size and attainment of critical weight in *Drosophila* larvae [12]. It is clear that in wild-type *Manduca*, the prothoracic gland size is not correlated with the critical weight. Thus, these findings further support the notion that the switch in response to starvation is distinctly regulated in *Manduca* and *Drosophila.* We observed that the prothoracic gland growth in rapamycin-fed *black* mutant larvae was not disproportionately affected relative to the body size. This curious effect might imply that the scaling relationship between the body size and the prothoracic gland size is coupled in the absence of JH.

### The *black* mutant larvae exhibit a bail-out response to starvation

In *Drosophila,* starvation after the critical weight leads to earlier onset of metamorphosis [10, 12]. These “bail-out mechanisms” have been observed in other insects, such as dung beetles that rely on ephemeral food supplies [28]. In the *black* mutant larvae, starvation post critical weight also led to earlier onset of metamorphosis, although the effect was only significant when larvae were fed rapamycin. Thus, in the absence of JH and insulin/TOR signaling, it appears that animals exhibit a bail-out response. We suggest that bail-out responses may be the default and that JH overrides these mechanisms to prolong the growth period in species where the costs of a small final body size outweigh the costs of delayed development.

The mechanism underlying this bail-out response is unknown at this time. We hypothesized that tissues in feeding larvae might grow and inhibit ecdysteroid release by secreting an inhibitory factor. In *Drosophila*, dILP8, secreted from growing imaginal discs, can delay the release of ecdysteroids from the prothoracic glands [23]. However, at least when wing imaginal discs were removed, the feeding larvae continued to delay metamorphosis relative to the starved larvae. This is perhaps not too surprising since *discless* mutant *Drosophila* also does not delay metamorphosis [29]. We therefore propose that some other factor, perhaps secreted from the fat body or other imaginal tissues besides wing discs, may be involved in the bail-out phenotype.

### TOR signaling is a component of the molt timer

Recently, a developmental timer was identified in which allatectomized larvae initiate metamorphosis after four days as long as larvae feed on amino acids for one day and even when the size of the larva is substantially below the critical weight [19]. Given that TOR signaling is an amino acid-sensitive pathway, it is likely a part of the timer. Our finding that feeding rapamycin to JH-deficient *black* mutants significantly increases the delay period indicates that TOR signaling likely contributes to the timer. Whether or not TOR signaling is the sole regulator of the timer remains to be seen.

## Conclusions

This study clarifies the distinct roles of JH and insulin/TOR signaling pathway in the determination of the final size of *Manduca*. Insulin/TOR signaling is important in the ecdysteroid biosynthesis/release, but its effect is only visible when JH titers are reduced. Furthermore, unlike in *Drosophila*, the critical weight in *Manduca* is independent of the insulin/TOR signaling pathway and likely depends solely on a size-sensing mechanism, possibly regulated by oxygen levels [7]. The mechanisms underlying the switch in the response to starvation clearly differ between *Manduca* and *Drosophila.* Thus, the operational definition of critical weight cannot be used interchangeably between different species. We suggest the use of the terms “nutrition-sensitive critical weight” and “size-sensitive critical weight” instead to distinguish between the two underlying mechanisms. As shown in Fig. 7, the relative times at which the two distinct threshold points are reached determine whether JH or insulin/TOR signaling dominates in the determination of the time to metamorphosis. The nutrition-sensitive critical weight is the time when the prothoracic gland becomes competent to secrete ecdysteroids and has grown, whereas the size-sensitive critical weight is the point at which the larva begins to clear JH. When this size-sensitive critical weight is reached early, nutrients will determine when ecdysone can be secreted. Currently, there is a debate about whether the critical weight in *Drosophila* (the nutrition-sensitive critical weight) is the same as the minimum viable weight, which is defined as the weight that the larva must reach in order to pupariate [2, 10, 27]. The question of whether or not the nutrition-sensitive critical weight and the minimum viable weight are identical concepts needs to be investigated in the future.

Despite the differences in critical weight, insulin/TOR signaling plays a major role in terminal growth period in both *Manduca* and *Drosophila*. This effect is predominantly masked by the presence of JH in the wild type but is revealed in the absence of JH. Thus, certain roles of insulin/TOR signaling are likely conserved between the two species, but the role of JH differs between the two species, altering the relative contribution of insulin/TOR signaling to the pattern of larval growth.

We propose that trade-offs between body size and developmental speed are mediated by levels of JH: JH serves to delay metamorphosis until a large enough size is attained, whereas in the absence of JH, attaining sufficient nutrition promotes the onset of metamorphosis (Fig. 7). This makes sense considering the life histories of *Drosophila* and *Manduca.* In nature, *Drosophila* feed on ephemeral food sources, such as rotting fruits, which run out during the course of development. In such cases, larvae must quickly undergo metamorphosis. Thus, they rely on nutrient-sensing mechanisms to determine the time to metamorphose. *Manduca,* in contrast, typically feed on solanaceous plants, which do not disappear during the course of larval development. Thus, in this species, a body size-sensing mechanism overrides the nutrient-sensing mechanism to delay metamorphosis until a sufficient size has been achieved. The existence of two distinct critical weights thus allows a population to adapt to maximize fitness in distinct ecological niches.

## Methods

### Animal husbandry

Wild-type *Manduca* were obtained from Carolina Biological. The *black* mutant strain was provided by Dr. Fred Nijhout (Duke University). The *Manduca* larvae were reared individually at 26 °C with a 18:6 h light:dark cycle. Larvae were fed either a normal diet or a non-nutritive diet, as described previously [18]. Larvae were given normal diet until head capsule slippage in the penultimate instar, at which point they were given non-nutritive diet for two days. Once the larvae molted to the final instar, experimental diets were given, and the cups housing the *Manduca* were wiped clean using a Kimwipe every three days.

### Rapamycin treatments

A portion of both strains of *Manduca* were fed rapamycin (LC labs), an inhibitor of the protein kinase target of rapamycin. In the fourth instar, treatment of diet with 1 mg of rapamycin per gram of diet leads to substantial delay in the timing of molting and a significant increase in the size at the end of the fourth instar [18]. A tenfold increase did not cause a further increase in the final body size, although the growth rate was decreased [18]. Thus, 1 mg of rapamycin per gram of diet was given to fifth instar larvae in this study. Rapamycin was dissolved in DMSO at a concentration of 0.5 g/mL and subsequently mixed in 1X phosphate buffered saline (0.02 M phosphate, 0.15 M NaCl, 0.0038 M NaH_2_PO_4_, 0.0162 M Na_2_HPO_4_; pH 7.4) to an effective concentration of 0.005 g/mL. For control animals, the same effective concentration of DMSO in PBS was used (1:9 solution of DMSO:PBS). When feeding the *Manduca* each animal received 5 g of food in a thin slice. Over the diet, 1 mL of either the DMSO or the rapamycin solution was spread evenly.

### Critical weight determination

To determine the critical weight, larvae were fed a nutritive diet until they reached a certain weight, at which point they were transferred to a non-nutritive diet. *Manduca* were randomly selected to be starved at a certain weight at the beginning of the time course. The starvation diets continued to be treated with either DMSO or rapamycin solution at the same concentration. Additional larvae were also preselected to be fed until gut purge. For both of these treatments, the time to gut purge was recorded, and the critical weight was identified using the classical critical weight determination method [5].

### Prothoracic gland measurements

The prothoracic glands of fed wild-type and *black* mutant *Manduca* on rapamycin or DMSO diet were dissected out from three animals once they reached a specific weight. These prothoracic glands were then fixed and imaged under a microscope as described previously [18]. Prothoracic gland area was measured using the software ImageJ.

### Analysis of growth trajectories

Animals were weighed daily at the same time each day to ensure each time point was 24 h apart. The peak size refers to the maximum body mass recorded for each animal. Growth rates were calculated by determining the slope of the linear phase of the larval growth. The terminal growth period was defined as the number of days taken for a larva at the critical weight to gut purge. KaleidaGraph (Synergy) was used to run ANOVAs and Student’s *t*-tests.

### Western blot analysis

Prothoracic glands, fat bodies, and wing discs from fifth instar DMSO- or rapamycin-treated larvae one day after feeding were used to extract proteins. The tissues were homogenized in NB Buffer (150 mM NaCl, 50 mM Tris–HCl pH 7.5, 2 mM EDTA, 0.1 % NP-40, 1 mM DTT, 10 mM NaF) with complete protease inhibitor cocktail (Roche, Germany) and PhosSTOP phosphatase inhibitor cocktail (Roche, Germany). After protein concentrations were quantified using a BCA assay, equal amounts of samples were loaded for SDS-PAGE. Anti-Phospho-Drosophila Akt (Ser505) (1:1000, Cell Signaling Technology), anti-Phospho-4E-BP1 (Thr37/46) (1:1000, Cell Signaling Technology), and anti-alpha tubulin (1:1000, Sigma) antibodies were used for western blot analyses.

### Imaginal disc extirpation

To determine whether the growth of wing imaginal discs in *Manduca* played a role in the “bail-out” response observed in starved *black* mutants post critical weight attainment, all four wing imaginal discs were removed from *black* mutant larvae on day 0 of the fifth instar. After the surgery, the open wounds in the cuticle were sealed using melted paraffin wax:peanut oil solution (in a 1:1 ratio). For the sham control, incisions were made in the areas of imaginal disc removal although the discs were not removed. Larvae were then fed a nutritive diet containing rapamycin until they reached 5–6 g. At this point, larvae were either transferred to a starvation diet with rapamycin or kept on the nutritive diet with rapamycin. Animals were weighed daily and checked for behavioral responses corresponding to gut purging.

### Sugar diet treatments

In order to explore the nutrition-based pathway for *black* mutant *Manduca sexta*, larvae were fed a nutritive diet containing rapamycin every two days until reaching 5–6 g, at which point they were placed on either a starvation or a sugar diet, which was made by adding 43 g/L sucrose to the non-nutritive diet. The larvae were fed and tracked daily until gut purge.
